# Identification of a Synergistic Multi-Drug Combination Active in Cancer Cells via the Prevention of Spindle Pole Clustering

**DOI:** 10.3390/cancers11101612

**Published:** 2019-10-22

**Authors:** Andrea Weiss, Morgan Le Roux-Bourdieu, Marloes Zoetemelk, George M. Ramzy, Magdalena Rausch, Daniela Harry, Marijana Miljkovic-Licina, Katayoun Falamaki, Bernard Wehrle-Haller, Patrick Meraldi, Patrycja Nowak-Sliwinska

**Affiliations:** 1 Institute of Pharmaceutical Sciences of Western Switzerland, Faculty of Sciences, University of Geneva, 1 Rue Michel-Servet, CMU, 1211 Geneva 4, Switzerland; Andrea.Weiss7@gmail.com (A.W.); Marloes.Zoetemelk@unige.ch (M.Z.); George.Ramzy@unige.ch (G.M.R.); Magdalena.Rausch@unige.ch (M.R.); 2 Translational Research Centre in Oncohaematology, 1 Rue Michel-Servet, CMU, 1211 Geneva 4, Switzerland; Morgan.LeRouxBourdieu@unige.ch (M.L.R.-B.); Marijana.Licina@unige.ch (M.M.-L.); Bernhard.Wehrle-Haller@unige.ch (B.W.-H.); Patrick.Meraldi@unige.ch (P.M.); 3 Department of Cell Physiology and Metabolism, University of Geneva Medical School, 1 Rue Michel-Servet, CMU, 1211 Geneva 4, Switzerland; Daniela.Harry@unige.ch (D.H.); Katayoun.Falamaki@etu.unige.ch (K.F.); 4 Department of Pathology and Immunology, University of Geneva Medical School, 1 Rue Michel-Servet, CMU, 1211 Geneva 4, Switzerland

**Keywords:** drug combinations, drug synergy, centrosome, multipolar spindle pole clustering

## Abstract

A major limitation of clinically used cancer drugs is the lack of specificity resulting in toxicity. To address this, we performed a phenotypically-driven screen to identify optimal multidrug combinations acting with high efficacy and selectivity in clear cell renal cell carcinoma (ccRCC). The search was performed using the Therapeutically Guided Multidrug Optimization (TGMO) method in ccRCC cells (786-O) and nonmalignant renal cells and identified a synergistic low-dose four-drug combination (**C2**) with high efficacy and negligible toxicity. We discovered that **C2** inhibits multipolar spindle pole clustering, a survival mechanism employed by cancer cells with spindle abnormalities. This phenotype was also observed in 786-O cells resistant to sunitinib, the first line ccRCC treatment, as well as in melanoma cells with distinct percentages of supernumerary centrosomes. We conclude that **C2**-treatment shows a high efficacy in cells prone to form multipolar spindles. Our data suggest a highly effective and selective **C2** treatment strategy for malignant and drug-resistant cancers.

## 1. Introduction

Combination therapies represent an attractive treatment option due to their potential to overcome drug resistance by targeting non-overlapping signaling pathways, thereby decreasing the probability of developing cross-resistance [1]. While off-target toxicities are a major concern of new drug combinations, synergistic drug combinations can achieve higher therapeutic selectivity than single drugs [2]. Causally, this can be explained by various phenomena. If properly designed, synergistic drug combinations can (i) enable targeting of deregulated networks from multiple angles [3], thereby challenging the robust and compensatory nature of complex biological systems [4,5], and (ii) allow for effective therapies with reduced toxicity by employing lower single drug doses of compounds with non-overlapping dose-related toxicities [6,7]. However, a major limitation of clinically used cancer drugs is the lack of specificity resulting in toxicity.

The use of properly tailored drug combinations is a promising alternative to overcome the above-mentioned limitations of cancer therapy. The limited success of clinically tested drug combinations, thus far, can largely be attributed to intolerable toxicities, lack of adequate efficacy and acquired drug resistance. This may be a result of current clinical practice where drug combination components are selected empirically or based on the success of single-drug therapies. Subsequently, the drugs combined are not selected for their synergistic or selective activity. To address these issues, various drug optimization methods have been developed [8,9]. One of the major challenges in defining optimal drug cocktails is the immense number of combinatorial possibilities. Not only must the selection of candidate drugs be taken into consideration, but also their relative dose ratios, their potential for synergisms or antagonisms, and their toxicity profiles. Already combining 10 drugs at six doses represents 6^10^, i.e., over 60 million, possible drug-dose ratio combinations. 

In order to solve these problems, we have previously developed the streamlined-Feedback System Control (s-FSC) technology, a strategy for rapid in vitro identification of optimized, synergistic low-dose multi-drug combinations [8,9,10,11]. In contrast to other approaches based on pharmacogenomics or high-throughput screening, this phenotypically-driven method identifies synergistic drug mixtures with minimal experimental effort. Moreover, it does not require background and mechanistic information about the system prior to the start of optimization. The s-FSC is based on the use of response surface methodology and an orthogonal array composite design (OACD) approach with linear regression analysis [8,9,12]. It selectively samples a minimal number of experimental data points in order to create the cell’s response surface, i.e., drug dose-efficacy dependence in terms of second-order linear regression models that are used to select synergistic drug interactions [9]. 

In this study, we used for the first time the Therapeutically Guided Multidrug Optimization (TGMO) method that is based on the s-FSC technique. In the TGMO approach, drug combinations are tested simultaneously on both cancer (in this case human clear cell renal cell carcinoma cell line, 786-O) and non-cancerous cells (e.g., nonmalignant renal cells, in this case HEK-293T cells) at low doses (i.e., below maximal plasma doses, MPD, in humans). Therefore, this strategy allows the identification of optimized multidrug combinations (ODC) acting with high efficacy and selectivity. We carried out our study in ccRCC, because it is the most aggressive and common type of kidney cancer, representing about 70% of kidney tumors [13]. This selection was also dictated by the chemo-and radio-resistant properties of ccRCC and the fact that the success of pharmacological intervention with kinase inhibitors (KI) and histone deacetylase inhibitors (HDACI) has been proven in the clinic. Moreover, many HDACI sensitize cancer cells to both cytotoxic and targeted agents [14]. Various clinical and preclinical data suggest synergistic interactions between KI and HDACI [15,16,17,18]. 

Starting from a set of four HDACI and six KI, using the TGMO method we identified a selective optimal drug combination (ODC) after testing 0.37% of all possible experimental conditions accompanied by modeling-based data analysis. The investigation on the mechanism of action of the ODC revealed the induction of cell cycle arrest, actin cytoskeleton disruption, and dysfunctional cell divisions due to persistent aberrant mitotic spindle structures.

## 2. Results

### 2.1. Therapeutically Guided Multidrug Optimization Screen Identifies Synergistic Low-Dose Drug Combinations Consisting of HDAC and Kinase Inhibitors

**Figure 1:**
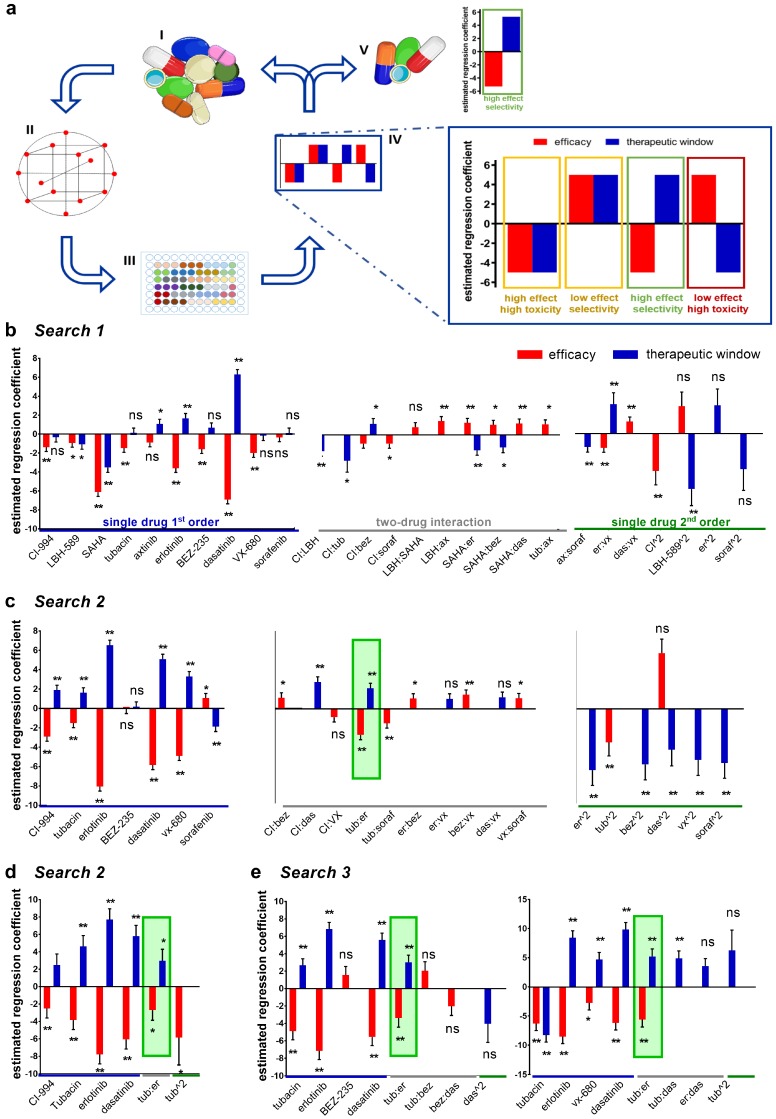
Therapeutically Guided Multidrug Optimization (TGMO)-based optimization of the drug combination. (**a**) Schematic overview of the TGMO-based optimization platform. (I) An initial set of ten drugs was selected. (II) Dose-response curves for each of ten drugs were generated for each cell type using inhibition of cell metabolic activity (ATP levels) as a measure for cell viability. The drug combination matrix to be tested was selected via an orthogonal array composite design (OACD). (III) The TGMO-based screen was initiated by experimentally testing a matrix of 91 drug combinations according to the drug combination matrix. (IV) The difference in drug efficacy between nonmalignant HEK-293T cells and malignant 786-O cells (in red) are defined as the therapeutic window (in blue). Regression coefficients of both the efficacy in 786-O cells and the therapeutic window were modeled using a step-wise second-order linear regression model. Orange frames indicate compounds showing not optimal drug interactions (either high efficacy with low toxicity or low efficacy with no toxicity), while compounds exhibiting an optimal activity profile (both effective and nontoxic) are framed in green. The red frame indicates compounds with poor activity profiles (lack of efficacy and considerable toxicity). (V) Data analysis allowed for drug selection, refinement of the searches and final the selection of the ODC. Estimated regression coefficients resulting from *Search 1* (**b**), *Search 2* (seven-drug and four-drug, **c** and **d**), and *Search 3* (**e**). Regression coefficients corresponding to models of efficacy in 786-O cells are represented in red and the therapeutic window models are presented in blue. Green boxes highlight the most relevant synergistic activity consistent throughout the sequential searches and resulted in the selection of the optimal combination. Significance is represented with * *p* < 0.05 and ** *p* < 0.01.

A set of ten compounds, which included four HDAC inhibitors, five tyrosine kinase inhibitors (TKI) inhibitors, and one serine-threonine KI (Table 1 and Supplementary Materials) was selected for the Therapeutically Guided Multidrug Optimization (TGMO)-based screen (Figure 1). 

The TGMO search was initiated by generating dose-response curves for each drug (Supplementary Figure S1). The experiments were performed in a simple in vitro cell viability bioassay (metabolic activity in ATP levels), using the human clear cell renal cell carcinoma (ccRCC) 786-O cell line and the nonmalignant human renal (HEK-293T) cells. 786-O cells were selected based on their lack of sensitivity to HDACI and known resistance to sunitinib, the first line treatment for ccRCC. The ED_50_ dose (the dose that inhibited 50% of cell viability, as compared to the control) of each drug was determined by fitting dose-response data on a semi-log scale. The ED_20_ (or lower, based on clinically attainable drug plasma concentrations) was selected as the highest dose for combinatorial drug screening (Table 1). The second drug dose used in the screen was half of the ED_20._

Drug combinations for experimental testing were defined by an orthogonal array composite design (OACD) [12] (Figure 1a,II and Supplementary Materials). This methodology defines a set of experimental points to be tested selected from all possible combinations. During the experiments (Figure 1a,III), the efficacy in the therapeutic window (TW, the difference in efficacy between HEK-293T and 786-O cells) was used to generate second-order linear regression models (Figure 1a,IV, efficacy in red bars and TW in blue bars, Supplementary Figure S2a). 

For each regression model, an estimated regression coefficient was generated for the significant terms in the model, including “single-drug first order”, “two-drug interaction”, and “single-drug second-order”. These terms describe the contribution of each drug as an individual agent and as a drug pair to the overall activity of the drug combinations. The accuracy and reliability of the regression models were assessed based on residual analysis (Supplementary Figure S2b). The generated models made it possible to analyze drug–drug interactions and to identify synergistic, selective drug interactions, guiding further drug selection and elimination. This facilitated refinement of the drugs to be tested in the subsequent experimental rounds and the selection of a drug combination with maximal cancer cell viability inhibition and minimal toxicity in noncancerous cells (Figure 1a,V). 

In *Search 1*, 91 drug combinations were screened. Stepwise regression analysis of the experimental data led to the elimination of three drugs, i.e., LBH-589, SAHA, and axitinib, due to the lack of synergistic interaction and/or no statistical significance in single drug first-order terms (Figure 1b). In *Search 2*, we tested a set of 50 combinations composed of up to seven drugs (Figure 1c,d) along with a set of 25 four-drug combinations (Figure 1e). The four-drug combinations selected based on *Search 1* were comprised of CI-994, tubacin, erlotinib, and dasatinib. *Search 3* (Figure 1e) evaluated other promising four-drug combinations identified in the seven-drug screen (*Search 2*). The synergy between tubacin and erlotinib was confirmed in all three of the four-drug sets investigated. The two four-drug sets investigated in *Search 3* did not show improved efficacy over the original four-drug combination screened in *Search 2*, as synergy was driven by the same interaction between tubacin and erlotinib and first-order effects were not superior to the primary selection. Thus, the TGMO method identified a selective ODC, from here on called **C1**, consisting of CI-994, tubacin, erlotinib, and dasatinib. 

The efficacy of **C1** was driven by a strong synergy between tubacin and erlotinib, as identified in the *Searches 2* and *3* (Figure 1b–e, highlighted in green), as well as by the additive contribution of erlotinib and dasatinib. The activity of **C1** showed highly selective and synergistic activity, as indicated by **C1** outperforming the corresponding monotherapies (*p* < 0.01) and by the lack of activity in the nonmalignant HEK-293T cell line (Supplementary Figure S3a). Response surfaces generated from the regression model of data obtained in *Search 2* (Figure 1e), demonstrated the synergistic interaction of tubacin and erlotinib (as evidenced by the slope of the surface), as well as the important contribution of all four compounds in the optimized combination (Supplementary Figure S3b).

**Figure 2:**
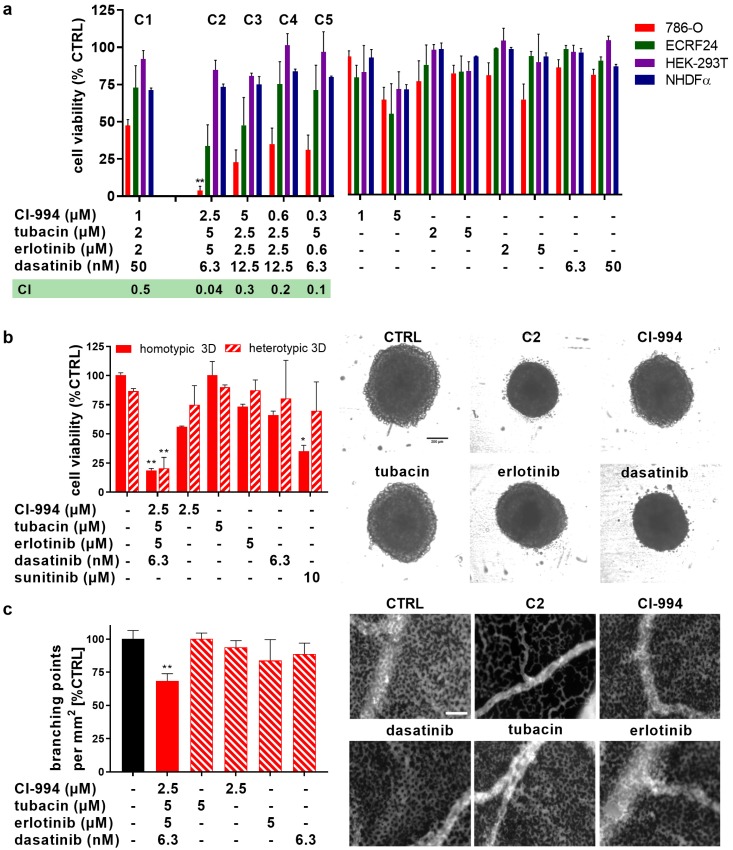
Dose optimization and validation of the OCD efficacy in 3D cell cultures with sunitinib-resistant cells and anti-angiogenic ODC potential in the chorioallantoic membrane model (CAM). (**a**) The efficacy of the five most promising drug combinations (**C1–C5**) derived from the dose optimization with **C1**, identifying **C2** as the most effective drug combination. Corresponding single drug treatments are presented for the 786-O cell line, non-malignant renal HEK-293T control cells, as well as in nonmalignant NHDFα fibroblasts and activated ECRF24 endothelial cells. Green box: the “combination index” (CI) values for each drug combination with CI < 1 indicating synergy (highlighted in green), 0 and CI > 1 indicating antagonism. * *p* < 0.05 and ** *p* < 0.01 represent significant increased activity of **C1** compared to **C2–C5** and corresponding single drug treatments as determined by a one-way ANOVA with post hoc Sidak’s multiple comparison test from N = 2–4 independent experiments. (**b**) Efficacy and representative images of the dose-optimized drug combination **C2** in 3D homotypic (786-O) spheroids or in 3D coculture heterotypic spheroids containing human fibroblasts, 786-O (1:1) and 10% ECRF24 endothelial cells. Sunitinib at 10 μM was used as a positive control. Scale bar represents 200 μm for all images. (**c**) In vivo inhibition developmental angiogenesis evaluated in the chorioallantoic membrane (CAM) model of the chicken embryo following two consecutive days of topical drugs administration. Fluorescence angiograms show the inhibition of capillary growth in CAM treated with **C2** as presented by the quantification of the number of branching points/mm^3^ based on the automated image-analysis. ** *p* < 0.01 represents significance versus CTRL as determined by a one-way ANOVA with post hoc Sidak’s multiple comparison test from N = 2 independent experiments (n = 4–15). Error bars represent ± SEM. Scale bar represents 800 µm.

In the final stage of the TGMO-based screen, *Search 4*, a broader dose range of these four compounds was considered and doses of compounds included in **C1** were optimized. 786-O cell viability inhibition after incubation with drug combinations **C2–C5** and corresponding monotherapies are presented in Figure 2a. **C2** inhibited 786-O cell viability by 96%, showing significantly greater activity than **C1** (*p* < 0.0071) and all single compound treatments. Drug combinations **C1–C5** were only minimally active in HEK-293T, as well as normal human fibroblast NHDFα cells, confirming the successful application of the therapeutic window-based drug optimization. Moreover, **C1–C5** also significantly outperformed the activity of non-optimal random drug combinations (Supplementary Figure S4), validating the TGMO-driven selection. The synergistic potential of each of the ODCs was further analyzed by calculating their respective Combination Indexes (CI) using Compusyn^©^ software [19]. While CI values lower than one signify synergistic drug combinations (highlighted in green), CI higher than one indicates antagonism and a CI between these values indicates additivity (Figure 2a). C2 showed over 10-fold higher synergy (CI = 0.04) than other ODCs and was hence selected for further evaluation. 

The activity of **C2** in cell viability inhibition was further tested in 3D homotypic (786-O cells) and 3D heterotypic (composed of 786-O cells, complemented with human NHDFα fibroblasts in ratio 1:1 and 10% activated human endothelial cells, ECRF24) cell culture models (Figure 2b). **C2** induced effective, approximately 80% cell viability inhibition in those models (*p* < 0.01 vs. CTRL and all monotherapies), confirming the results obtained in the 2D cell cultures (Figure 2a). 

Since anti-angiogenic treatment may potentiate the overall treatment of ccRCC, we exposed human immortalized ECRF24 endothelial cells to **C1–C5** and corresponding monotherapies. Interestingly, while **C2** and **C3** were virtually inactive in NHDFα cells, they were quite potent in activated human endothelial cells ECRF24 cells. These drug combinations induced inhibition (66% and 52%, respectively) of ECRF24 cell viability. Treatment with **C2** also led to increased apoptosis induction in endothelial cells (12.6% vs. 1.6% in CTRL, Supplementary Figure S5a), which was mainly driven by tubacin, but did not influence endothelial cell migration in 2D (Supplementary Figure S5b) or 3D network formation (Supplementary Figure S5c). The anti-angiogenic potential of **C2** was further confirmed in the in vivo developmental chorioallantoic membrane (CAM) model [20,21], where **C2** significantly (*p* < 0.0001) reduced the development of the microvasculature (quantified by the number of branching points/mm^2^, Figure 2c). These results suggest therefore a potential dual, i.e., anticancer and angiostatic, mechanism of action of **C2**.

### 2.2. **C2** Activity on Cell Cycle Regulation, Actin Cytoskeleton Reorganization, and Nuclear Structures

In order to investigate the potential mechanism of action of **C2**, we monitored cell cycle distribution and the frequency of apoptosis with propidium iodide staining using flow cytometry. Exposure of 786-O cells to **C2** for 24 h resulted in higher apoptosis levels (8.1% vs. 1.1% in CTRL, *p* = 0.0109, Supplementary Figure S6a, top graph). After 72 h of drug incubation, the higher incidence of apoptosis was no longer observed, however, a G2/M phase arrest was registered (12.3% and 21.3% for CTRL and **C2**, respectively, *p* = 0.0083, Supplementary Figure S6a bottom graph). Sunitinib at a concentration of 10 µM was used as positive control. 

Morphological changes in **C2** treated cells were also investigated based on fluorescent phalloidin (f-actin marker) and nuclear DAPI (4′,6-diamidino-2-phenylindole) staining. After **C2** treatment, we observed changes in the actin cytoskeleton morphology. The cells presented enhanced stress fibers in the center, but also the formation of f-actin cables running along the cell borders (Supplementary Figure S6c, white arrows). Nevertheless, **C2** did not affect 786-O migration in a 2D migration assay (Supplementary Figure S7). DAPI staining also indicated that **C2** treatment led to a three-fold increase (7.5% ± 1.3%) in the number of micronuclei vs. CTRL treatment (1.8 ± 0.3%, *p* < 0.001, Supplementary Figure S6b,c, red arrow). Micronuclei induction is a marker for chromosome segregation errors or chromosome aberrations, known to be linked to genomic instability and chromosomal damage [22]. The same treatment also induced other significant nuclear abnormalities (Supplementary Figure S6c, yellow arrow), mostly driven by CI-994. The nuclear abnormalities and the G2/M enrichment in the FACS profile suggested potential defects in cell division, such as chromosome segregation errors (micronuclei) or cytokinesis failure (multi-nuclei) [23].

### 2.3. **C2** Prevents the Clustering of Multipolar Spindles in 786-O Cells

**Figure 3:**
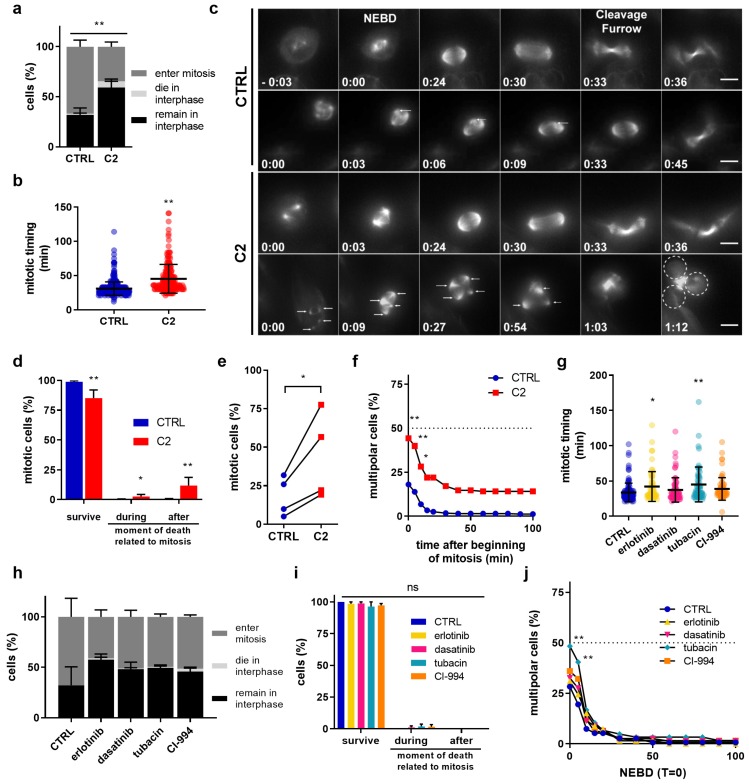
**C2** prevents centrosome clustering in 786-O cells during metaphase. (**a**) Outcomes of 786-O cells treated with CTRL (0.1% DMSO) or **C2** during 24 h movies. ** *p* < 0.0001 vs. CTRL in a Fisher’s exact test, N = 4 experiments with n = 606 (CTRL) and n = 603 (**C2**) cells (**b**) Mitotic timing is defined as the time between nuclear envelope breakdown (NEBD) and cleavage furrow formation. ** *p* < 0.001 vs. CTRL in a Mann–Whitney Test from N = 4 independent experiments with a total of n = 206–406 cells. (**c**) Time-lapse images of CTRL (0.1% DMSO) or **C2**-treated 786-O cells undergoing mitosis, stained with SiR-Tubulin. Mitotic timing in h:mins using nuclear envelope breakdown (NEBD) as T = 0. The arrows in the **C2**-treated multipolar cells indicate spindle poles, dashed circles the daughter cells after mitotic completion. Scale bar = 10 µm. (**d**) Mitotic outcomes of 786-O cells treated with CTRL (0.1% DMSO) or **C2**. ** *p* < 0.0001 and * *p* < 0.05 vs. CTRL in a Fisher’s exact test, N = 4 independent experiments with n = 406 (CTRL) and n = 206 (**C2**) cells. (**e**) Percentage of multipolar cells in individual experiments. * *p* < 0.05 vs. CTRL in paired two-tailed T-test from N = 4 independent experiments; n = 406 (CTRL) and 206 (**C2**) cells. (**f**) Percentage of multipolar cells over time. ** *p* < 0.005 and * *p* < 0.05 for the first three points compared to CTRL in a two-way ANOVA with Sidak’s multiple comparisons test from N = 4 independent experiments with n = 406 (CTRL) and n = 206 (**C2**) cells. (**g**) Mitotic timing of 786-O cells treated with the single drugs used for **C2**. Significances of ** *p* = 0.0008 and * *p* = 0.0236 vs. CTRL group in a two-way ANOVA with Tukey’s multiple comparisons test from N = 4 experiments with n = 62–123 cells. (**h**) Outcomes of 786-O cells treated with CTRL or monotherapies during 24 h movies. N = 4 experiments with n = 123–160 cells. (**i**) Mitotic outcome of 786-O cells treated with CTRL (0.1% DMSO) or monotherapies. None of the monotherapies was significantly different compared to CTRL group in a two-way ANOVA with Tukey’s multiple comparisons test from N = 4 experiments with n = 62–123 cells. (**j**) Percentage of multipolar cells over time. ** *p* < 0.01 for the first two points of tubacin treated cells vs. CTRL in a two-way ANOVA with Sidak’s multiple comparisons test from N = 4 experiments with n = 62–123 cells. Error bars represent SEM for all graphs.

To test whether **C2** treatment affects cell division, we monitored cell proliferation by live-cell imaging. 786-O cells were treated with 0.1% DMSO in the cell culture medium (CTRL) or **C2** and stained with the live-cell dye SiR-tubulin (microtubule marker) to label the mitotic spindle, monitor mitotic progression, and detect any cytokinesis failure. All experiments were performed in the presence of Valspodar (4 µM), a multidrug pump inhibitor that prevented SiR-tubulin removal from the cell (this concentration of Valspodar had no effect on the efficacy of **C2**). Live cells were recorded for 24 h at a temporal resolution of 3 min. We found that during these 24 h, fewer **C2** treated cells entered mitosis compared to CTRL (35% vs. 66%) and more of them died in interphase before any mitotic entry (6% vs. 1%; Figure 3a), pointing to a cell cycle delay. Furthermore, **C2**-treated cells exited mitosis later, as the mean time between nuclear envelope breakdown (NEBD, time point at which the spindle is set up) and the appearance of the cleavage furrow increased from 30 ± 10 min in CTRL-treated cells to 42 ± 6 min (Figure 3b,c). Importantly, a significantly higher number of **C2**-treated cells died during mitosis (3% vs. 0% in CTRL) or after mitotic exit in the ensuing interphase (12% vs. 0.5% in CTRL, Figure 3d). While our analysis did not reveal any cytokinesis defect, we frequently found multipolar spindles in **C2** treated cells (44% vs. 18% in CTRL-treated cells, Figure 3e). 

Such multipolar spindles can arise in the presence of extra centrosomes, the main microtubule organizing centers in mitosis [24], or can be caused by fragmentation of the pericentriolar material (PCM). Persistent multipolar spindles result in multipolar cell divisions which in turn frequently leads to cell death in the ensuing interphase due to massive chromosome mis-segregations [25,26]. Cancer cells possess a survival mechanism to overcome this problem called spindle pole or centrosome clustering, where supplementary spindle poles are gathered together to form a pseudo-bipolar spindle, a phenomenon that was also visible in 786-O cells (Figure 3c, CTRL condition, white arrows). We, therefore, plotted the percentage of multipolar spindles over time and found that **C2** treated cells were severely delayed in their ability to cluster multipolar spindles compared to the CTRL, resulting in 15% of the cells failing to resolve their multipolar spindles over the entire course of mitosis (Figure 3c,f). Importantly, these mitotic phenotypes were specific for the **C2**-treatment, as apart from a small mitotic delay in cells treated with erlotinib or tubacin, no equivalent phenotype could be observed in cells treated with the single components of the **C2** combination (Figure 3g–j).

As an additional control, we also tested the effects of **C2** on HEK-293T cell division, as this cell line had not shown a large decrease in viability after **C2** treatment (Figure 2a). Live cell imaging performed on HEK-293T cells showed that even though **C2** prolonged mitotic timing and reduced the number of cells entering mitosis (Supplementary Figure S8a–c), it did not increase the proportion of cells with multipolar spindles, unlike what has been observed in 786-O cells (Supplementary Figure S8d and Figure 3f). Moreover, no major cell death was observed in interphase (1.5% vs. 0% in CTRL), during mitosis (2% vs. 0% in CTRL) or after mitotic exit in the ensuing interphase (1% vs. 0% in CTRL) (Supplementary Figure S8c,e). This suggested that **C2** specifically induces cell death in 786-O by prolonging multipolarity during mitosis. 

### 2.4. **C2** Activity Prevents Spindle Pole Clustering in Sunitinib-Resistant 786-O Cells

**Figure 4:**
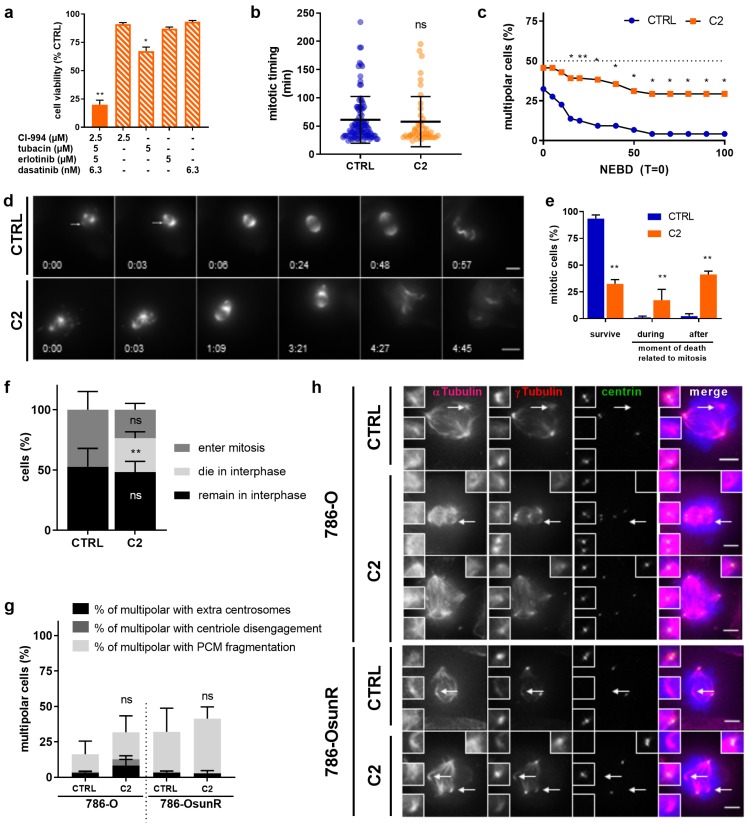
**C2** activity also prevents spindle pole clustering in sunitinib-resistant 786-O cells. (**a**) Efficacy of **C2** and corresponding single drug treatments in 786-O cells chronically exposed to sunitinib treatment (786-OsunR). ** *p* < 0.0001 and * *p* < 0.05 represent significances versus all corresponding single drug treatments from N = 5 independent experiments as determined by a one-way ANOVA with post-hoc Tukey’s multiple comparison test. (**b**) Mitotic timing of 786-OsunR treated with CTRL (0.1% DMSO) or **C2**. *p* = 0.2262 compared to CTRL as determined by a Mann–Whitney Test from N = 3 experiments with n = 65–106 cells. (**c**) Percentage of multipolar cells over time. * *p* < 0.05 and ** *p* < 0.005 vs. CTRL group in a two-way ANOVA with Sidak’s multiple comparisons test from N = 3 experiments with n = 65-106 cells for CTRL and **C2**-treated cells. (**d**) Time-lapse images of CTRL and **C2**-treated 786-OsunR cells stained with SiR-Tubulin. Mitotic timing in h:mins using NEBD as T = 0. Arrows in the CTRL multipolar 786O-sunR cells indicate spindle poles. Scale bar = 10 µm (**e**) Mitotic outcome of 786-O-sunR cells. ** *p* < 0.0001 vs. CTRL in a Fisher’s exact test, N = 3 experiments with n = 106–65 cells for CTRL and **C2**-treated cells. (**f**) Outcomes of 786-OsunR cells treated with CTRL or **C2** during the 24 h movie. ** *p* < 0.0001 vs. CTRL in Fisher’s exact test, N = 3 experiments with n = 221 and n = 259 cells (**g**) Percentage of fixed multipolar cells with extra centrosomes, disengaged centrioles or fragmented pericentriolar material (PCM) after 12 h of CTRL or **C2** treatment. Within each cell line, **C2**-treated cells were compared to CTRL cells in Fisher’s exact test from N = 3 experiments with n = 112–164 cells. (**h**) Representative immunofluorescence images of 786-O and 786-OsunR cells, treated for 12 h with CTRL or **C2,** stained for α-tubulin (magenta), γ-tubulin (red), centrin (green), and DAPI (blue). Scale bar = 5 µm. White arrows indicate poles formed by PCM fragmentation. Error bars represent SEM in all graphs.

The first-line therapy for advanced ccRCC is sunitinib, a tyrosine kinase inhibitor to which patients rapidly develop resistance. Therefore, we evaluated whether our optimized drug combination **C2** would also be effective in sunitinib-resistant cells (786-OsunR). First, 786-O cells were chronically exposed to sunitinib treatment (1 µM) and dose-response curves were generated to validate resistance induction (Supplementary Figure S9a). Moreover, intracellular and intralysosomal accumulation, one of the resistance mechanism of sunitinib [27], was observed (Supplementary Figure S9b). **C2** effectively inhibited the viability of 786-OsunR cells and significantly outperformed all single drug treatments (Figure 4a, *p* < 0.01 vs. CTRL and all monotherapies). 

To determine whether **C2** also impaired cell division in 786-OsunR cells, we performed live cell-imaging experiments after CTRL or **C2** treatment. While **C2** does not affect mitotic timing (Figure 4b), it prevented spindle pole clustering into a bipolar spindle over the course of mitosis in the 786-OsunR cells (30% vs. 5% for CTRL; Figure 4c,d). Moreover, **C2** treatment led to a higher incidence of cell death during mitosis (17% vs. 1% in CTRL) and after mitotic exit (41% vs. 2% in CTRL; Figure 4e). We conclude that **C2** prevented bipolar spindle formation in a large segment of the 786-OsunR cells and led to mitotic cell death independent of sunitinib-induced resistance [28]. Nevertheless, we note that **C2** treatment led to a high proportion of cell death in interphase before cells could enter mitosis, implying that **C2** also has a mitosis-independent mechanism of action in the 786-OsunR cells (Figure 4f). 

Multipolar spindles can arise via different mechanisms: fragmentation of the pericentriolar material, premature splitting of the two centrioles within the centrosomes, or presence of more than two centrosomes, as is frequently the case in cancer cells [29]. 786-O cells have been reported to only have a low percentage of cells with more than two centrosomes [30]. We therefore investigated the nature of the multipolar spindles in 786-O and 786-OsunR cells by immunofluorescence. Cells were synchronized in mitosis with the microtubule-depolymerizing drug nocodazole, released from this treatment to allow them to form a spindle and stained for α-tubulin (spindle marker), γ-tubulin (pericentriolar material marker), and centrin (centriole marker). Our analysis revealed that most multipolar spindles in both cell lines treated with or without **C2** resulted from the fragmentation of the pericentriolar material (Figure 4g,h).

### 2.5. **C2** Treatment Shows Greater Efficacy in the Cells with Abnormal Centrosome Numbers

**Figure 5:**
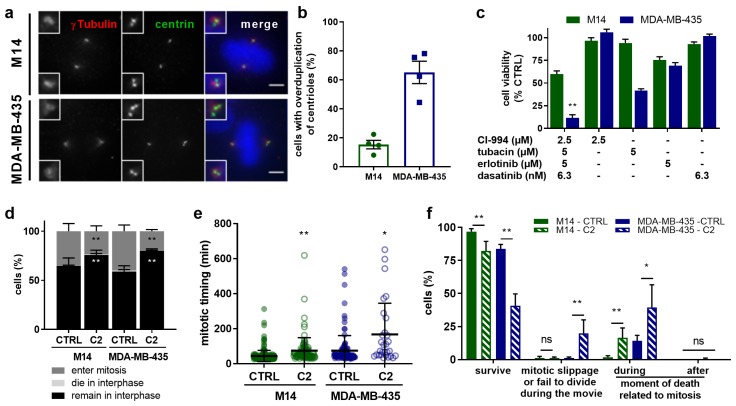

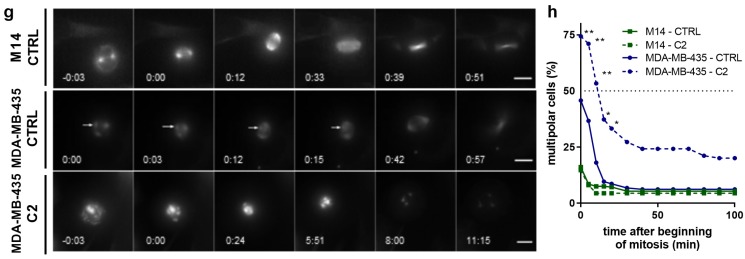
Effects of **C2** treatment on melanoma cells with normal or elevated number of centrosomes. (**a**) Representative images of M14 and MDA-MB-435 cells stained for centrin (green), γ-tubulin (red), and DAPI (blue). Scale bar = 5 µm. (**b**) Image-based quantification of the number of centrioles per cells. Results from N = 4 experiments with n = 165 cells for M14 and 164 cells for MDA-MB-435 cells. (c) Efficacy of **C2** and corresponding single drug treatments in M14 and MDA-MB-435 cells, ** *p* < 0.01 versus all corresponding single drug treatments in a one-way ANOVA with posthoc Tukey’s multiple comparison test. (**d**) Outcomes of M14 and MDA-MB-435 cells treated with CTRL (0.1% DMSO) or **C2** during 24 h movies. ** *p* < 0.0001 vs. CTRL in a Fisher’s exact test, N = 3 experiments, n = 610–515 cells for M14, and n=355-297 cells for MDA-MB-435 cells. (**e**) Mitotic timing from NEBD until formation of a cleavage furrow in min. ** *p* < 0.0001 for M14 cells and * *p* = 0.0002 for MDA-MB-435 cells, (CTRL vs. **C2**) in a Mann–Whitney Test from N = 3 experiments with n = 227–122 cells for M14 and n = 140–60 cells for MDA-MB-435. (**f**) Mitotic outcome of M14 and MDA-MB-435 cells. ** *p* < 0.0001 and * *p* < 0.001 vs. CTRL in a Fisher’s exact test, N = 3 experiments with n = 227–122 cells for M14 and n = 140–60 cells for MDA-MB-435. (**g**) Time-lapse images of M14 and MDA-MB-435 cells undergoing mitosis, stained with SiR-Tubulin. Mitotic timing in h:mins using nuclear envelope breakdown (NEBD) as T = 0. Arrows indicate spindle poles in the **C2**-treated multipolar MDA-MB-435 cells. Scale bar = 10 µm. (**h**) Percentage of multipolar cells over time. * *p* < 0.05 and ** *p* < 0.01 vs. CTRL in a two-way ANOVA with Sidak’s multiple comparisons test from N = 3 experiments with n = 227–122 (CTRL) and n = 140–60 cells (**C2**) M14 and MDA-MB-435 cells. Error bars represent SEM in all graphs.

Based on our results, we hypothesized that one of the main mechanisms by which **C2** generally kills cancer cells is by preventing spindle pole clustering. To strengthen this hypothesis we tested whether **C2** can also target cancer cells from other tissues displaying elevated centrosome/centriole numbers, which should be prone to form multipolar spindles. We evaluated its effects on two melanoma cell lines that have been reported to either have low (M14) or abnormally high (MDA-MB-435) levels of centrosome/centriole numbers [30]. Consistent with these reports, we found that 65% of MDA-MB-435 had more than four centrioles (vs. 15% in M14 cells; Figure 5a,b). Interestingly, **C2** inhibited cell viability much more efficiently in MDA-MB-435 than in M14 cells (90 ± 10%, vs. 40 ± 11%; Figure 5c). Live-cell imaging indicated that the **C2** treatment resulted in fewer cells entering mitosis and a mitotic delay for both cell lines (Figure 5d,e), matching our results in 786-O, 786-OsunR, and HEK-293T cells. During mitosis, more MDA-MB-435 cells failed to divide properly and died in mitosis after **C2** treatment compared to M14 cells (Figure 5f). Moreover, **C2** treatment resulted in an elevated number of persistent multipolar spindles in MDA-MB-435 cells, but not in M14 cells (Figure 5g,h). 

**Figure 6:**
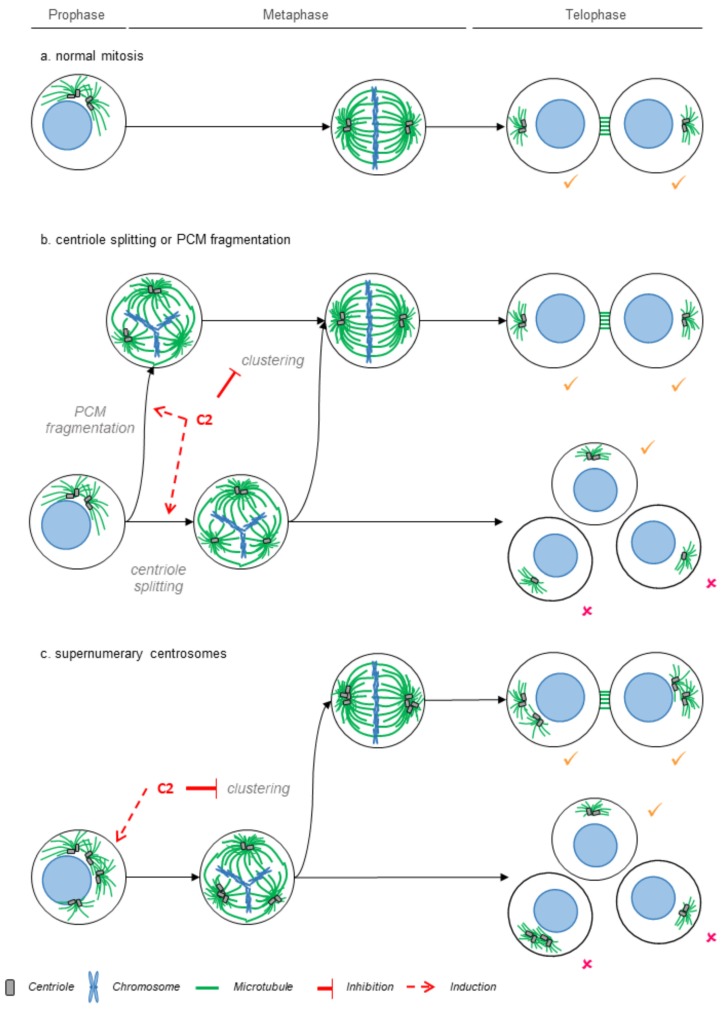
Model for the effects of the **C2** combination on cell division. (**a**) Faithful chromosome segregation requires the formation of a bipolar spindle (green) that segregates the genetic material into two daughter cells (normal mitosis). Cancer cells are prone to form multipolar spindles due to fragmentation of the pericentriolar material or premature centriole splitting (**b**) or due to the presence of elevated centrosome numbers (**c**). Since multipolar spindles are often lethal, cancer cells rely on a spindle pole clustering mechanism to form pseudo-bipolar division for survival. Our results are consistent with the hypothesis that the **C2** treatment prevents spindle pole clustering and potentially induces multipolar spindle formation, thus increasing the incidence of lethal multipolar cell divisions.

These results were consistent with our hypothesis and implied that the efficacy of the **C2**-treatment is linked to the propensity of cancer cell lines to form multipolar spindles due to abnormal centrosome/centriole numbers (MDA-MB-435) or fragmentation of the pericentriolar material (786-O); see schematic model in Figure 6.

## 3. Discussion

In this study, the phenotypically-driven TGMO-based screen was used to identify an optimal low-dose drug combination for ccRCC treatment with high efficacy and specificity towards a malignant clear cell-renal cell carcinoma 786-O cell line. This was achieved after the screening of only a small fraction of possible drug combinations in the entire search space of the drugs included in the screen. To increase specificity towards malignant cells, we introduced further constraints to our previously developed s-FSC method, which included (i) the parallel experimental testing in malignant and non-malignant cells to create opportunities for the therapeutic window (TW) and (ii) the inclusion of drug doses in the range of clinically attainable drug plasma concentrations, leading to the TGMO approach. Consequently, this resulted in the input drug dose of certain compounds (axitinib, VX-680, and sorafenib) that were even lower than the targeted ED_20_ [8]. Although counterintuitive to the optimization criteria, this reflects the heterogeneous treatment responses of different cell lines to clinically used drugs and the lack of efficacy of certain individual drug treatments at clinically relevant doses [31]. Modeling analysis of both efficacy (786-O cell viability inhibition) and the TW (difference between nonmalignant and malignant cells) led to the identification of a 786-O cell-specific and synergistic optimal drug combination (ODC) composed of CI-994, tubacin, erlotinib, and dasatinib (drug combination **C2**). **C2** exhibited superior activity as compared to the current first-line standard ccRCC treatment (sunitinib).

When analyzing the regression models derived from the screen, the generated first-order terms clearly confirmed differences in mechanism and selectivity of the different drug classes included in the screen, i.e., HDACI and KI. While HDACI tended to have negative regression coefficients for efficacy in cancer and noncancerous cells, indicating a lack of selectivity towards malignant cells, KI generally gave positive regression coefficients in the TW indicating selective activity in cancer cells. Using the TW as an objective parameter for optimization, together with constraints on the drug doses limited by the maximally attainable clinical plasma drug concentrations, we identified a selective ODC with minimal activity in cross-validated nonmalignant cells.

The dose optimization step (*Search 4*) using both drug activity and the TW allowed for further increased efficacy and selectivity. These results support previous observations that in drug combinations, enhanced selectivity requires the use of multiple compounds (more than two) at optimized doses [32]. It has been proposed that the enhanced selectivity observed between synergizing drugs largely results from multitarget interactions, where one would expect synergies to be present in a relatively narrow spectrum of cellular phenotypes, increasing the chances of developing effective combinations without compromising the safety profile [2]. We proved that we can actively select a phenotype to be targeted, in our case the viability of cancer cells. Indeed, **C2** optimized for cell viability inhibition did not necessarily present enhanced activity in the cell migration inhibition or endothelial network formation although it did show anti-angiogenic properties when tested in a developmental angiogenesis assay (i.e., the chicken embryo chorioallantoic membrane assay). Identified drug interactions included both antagonisms and synergies, guiding the selection of the most synergistic and potentiating drugs for the final optimized drug combination. Further refinement of the drug selection through *Search 2* and *Search 3* allowed for the confirmation of robust drug interactions, in particular the synergy between tubacin and erlotinib (Figure 1c–e). This is in line with the results of Liu et al. (2012) who demonstrated that HDAC6 treatment with trichostatin A or tubacin can effectively downregulate the activity of EGFR in the pdkd1-mutant epithelial cells, which in turn, results in the decreased phosphorylation of other targets downstream of EGFR, including ERK1/2 [33]. Furthermore, the VHL status of 786-O cells might also play a role in the synergy between erlotinib and tubacin in 786-O cells. The loss of VHL has been shown to extend the activation of EGFR and is associated with increased receptor half-life and retention in the endocytic pathway (the half-life time of EGFR is approx. 1.5 h in 786-O cells with VHL reconstitution and greater than 4 h in 786-O cells treated with TGF-α or EGF) [34], thus creating an important EGFR-dependent survival and growth advantage. These findings connect HDAC activity with EGFR inhibition and may partially explain the synergy between tubacin and erlotinib sensitizing the cells to apoptosis induction. 

Acquired resistance to sunitinib is a common occurrence in the treatment of ccRCC [35,36]. It has recently been described that higher-order drug combinations may be needed to effectively target drug-resistant cells [37], which is further supported by the fact that the 786-O-specific **C2** consists of four compounds. Importantly, **C2** retained its anticancer efficacy in 786-OsunR cells, thus evidencing its potential to overcome cross-resistance mechanisms. This may suggest the need for higher-order drug combinations in targeting resistant cell types.

Based on our phenotypic analysis we postulate that the selective and synergistic anticancer efficacy of **C2** in 786-O cells and MDA-MB-435 is based in part on its ability to favor persistent multipolar spindles. This is due to its ability to prevent spindle pole clustering, but the **C2** treatment might also induce multipolar spindles on its own. Compounds that prevent pole clustering have emerged as a promising therapeutic agent, since they specifically target cancer cells that form multipolar spindles due to abnormal centrosome numbers, but do not affect mitotic progression in nonmalignant cells with normal centrosome numbers. Indeed, while normal cells as a rule have at mitotic onset 2 centrosomes with 2 centrioles, cancer cells frequently display abnormalities in centrosome organization, such as elevated centrosome numbers [26], which will impose transient multipolar spindles [38]. The minus-end directed kinesin HSET/KIF1C was the first promising target studied to develop new anticlustering drugs. As HSET depletion prevents clustering, it efficiently targets cancer cells with supernumerary centrosomes [39,40], yet it also induced multipolarity with acentrosomal poles [41]. So far, three HSET inhibitors have been reported: CW069, AZ82, and SR31527 [42,43,44], but their clinical efficacy has not yet been reported. We note that the phenotypes we observe with **C2** treatment closely mirror the effects seen after HSET depletion. Indeed, **C2** treatment prevents spindle pole clustering, and it might favor the formation of multipolar spindles, as at the first time point of our live cell imaging movies, we see more multipolar spindles. Alternatively, the lower percentage of initial multipolar spindles in DMSO-treated cells could be explained by rapid clustering within the first three minutes after NEBD. 

We hypothesize that **C2** targets cells prone to form multipolar spindles both in the context of abnormal centrosome/centriole numbers (MDA-MB-435) or in case of pericentriolar material fragmentation (786-O). This hypothesis is strengthened by our observation that **C2** selectively targeted MDA-MB-435 cells, but not M14 cells, which generally have a normal set of centrosomes. Indeed, while elevated centrosome numbers are associated with mild chromosomal instability and cancer progression, persistent multipolar spindles with or without centrosomes are associated with massive chromosome segregation errors and cell death [45,46,47,48,49]. Moreover, we note that **C2** treatment was also associated with micronuclei formation, which is prone to chromothripsis and loss of genetic information [50]. Nevertheless, we cannot exclude that apart from the ability to form multipolar spindles, other genetic differences between M14 and MDA-MB-435 cells play a role in determining the selective efficacy of the **C2** treatment. 

In particular, we note that in 786-O and 786-OsunR cells, **C2** triggered cell death not only during mitosis, but also before cells could divide (Figure 3 and Figure 4). This indicates that **C2** also targets cancer cells via a mitosis-independent mechanism. Several declustering agents have been shown to target cells both in interphase and mitosis, yet their mechanism of action focuses on the disruption of the nuclear-centrosome-Golgi axis, which inhibits cell migration [51]. Since **C2** did not affect cell migration, further investigation will be required to understand the **C2** mechanism of action in interphase. We speculate that HDAC6 inhibition could play a critical role, as this enzyme is involved in ciliary disassembly [52], a mechanism causing cellular senescence when disrupted [53].

## 4. Materials and Methods

### 4.1. Compounds

CI-994, LBH-589, SAHA, axitinib, erlotinib HCl, BEZ-235, dasatinib, and sorafenib were purchased from LC Labs (Woburn, MA, USA), tubacin (SML0065) was purchased from Sigma-Aldrich (St. Louis, MO, USA) and VX-680 from Selleck Chemicals (Houston, TX, USA). 

Compounds were dissolved in sterile DMSO (Sigma-Aldrich) at the following concentrations: CI-994 (20 mg/mL), LBH-589 (5 mg/mL), SAHA (5 mg/mL), tubacin (5 mg/mL), axitinib (20 mg/mL), erlotinib HCl (15 mg/mL), BEZ-235 (1 mg/mL), dasatinib (5 mg/mL), VX-680 (20 mg/mL), and sorafenib (40 mg/mL). Aliquots were stored at −80 °C and thawed prior to each experiment. A maximal concentration of 0.1% DMSO was allowed for any of the screened conditions and was used as a control (CTRL). 

### 4.2. Cells

Human renal cell carcinoma cell line 786-O and human embryonic noncancerous HEK-293T cells were purchased from ATCC. Normal human dermal fibroblast adult (NHDFα) cells were purchased from Lonza. The M14 and MDA435 cells were kindly gifted by Dr. M. Bettencourt-Dias. Immortalized ECRF24 endothelial cells macrovascular human endothelial cells immortalized (via immortalization procedures with amphotrophic replication-deficient retrovirus as described in [54]. Culture media consisted of RPMI for 786-O and HEK-293T cells, and DMEM for NHDFα, M14 and MDA435 cells, supplemented with 10% of 5% fetal bovine serum and 1% penicillin/streptomycin (Life Technologies, Carlsbad, CA, USA). Cells have recently been tested for mycoplasma contamination and have been authenticated with PCR. 

Human umbilical vein endothelial cells (HUVEC) were isolated from human umbilical cords and used for experiments only until passage 4. HUVEC cells were maintained in complete M199 medium (Gibco by ThermoFischer Scientific, Reinach, Switzerland) prepared freshly every week, supplemented with 10% Fetal Bovine Serum (FBS), 1% penicillin/streptomycin (ThermoFischer Scientific), 1% Endothelial Cell Growth supplement (ECGS; Millipore), 0.1 mg/mL heparin sodium salt (Sigma-Aldrich), 0.1 µM Hydrocortisone (Sigma-Aldrich), and 10 µg/mL L-ascorbic acid (Stock 10 mg/ml, dilute 1:1000, Sigma-Aldrich). HUVECs were grown in cell culture flasks coated with 0.2% gelatin (Sigma-Aldrich) and 1 mg/mL Collagen G (Biochrom AG, Berlin, Germany) in phosphate-buffered saline (PBS). Human vascular pericytes were purchased from ScienCell Research Laboratories and cultured according to the manufacturer’s instructions. Pericytes were cultured in Pericyte Medium (ScienCell Research Laboratories, Carlsbad, CA, USA) supplemented with 2% FBS, 1% Pericyte Growth Supplement (ScienCell Research Laboratories), and 1% penicillin/streptomycin solution (ScienCell Research Laboratories). The cells were grown in cell culture flasks coated with 15 µg/mL poly-L-lysine (Chemie Brunschwig AG, Basel, Switzerland) in H_2_O.

786-OsunR cells were generated by chronically exposing ccRCC cell lines to sunitinib treatment (1 µM). The response of cells to treatment was experimentally verified every two weeks. Cells were considered to show phenotypic resistance when the response of chronically exposure to sunitinib treatment was significantly reduced for at least one drug dose at or above the chronically administered dose. 

### 4.3. Cell Viability

For cell viability experiments, cells were seeded in 96-well culture plates and 72 h treatments with 0.1% DMSO CTRL or drug (combinations) were initiated 24 h post-seeding. Seeding density was 2–10 × 10^3^ cells/well, depending on the cell line’s growth characteristics such that the control cells approach confluence at the time of the assay readout. Subsequently, cell viability was assessed using the CellTiter-Glo^®^ luminescence assay (Promega, Madison, WI, USA) and cell viability was reported as the luminescence signal in the treated wells normalized to the signal in the DMSO treated control wells [55]. Dose-response curves and IC_50_ values were determined in Graphpad Prism^®^ using a four-parameter nonlinear fit of the log-transformed dose data of each compound.

### 4.4. Data Modeling and Analysis for Combinatorial Drug Screening

The TGMO platform was applied as previously described for s-FSC [8]. Briefly, drug combinations were selected based on a design of experiment (DoE) using the orthogonal array composite design (OACD) [12]. The activity of each drug combination was experimentally defined *in vitro* cell metabolic activity assays using therapeutic window and including maximal tolerated dose of each drug in human (Supplementary Figure S2a) and results were modeled in Matlab^®^ using second-order step-wise linear regression models. The accuracy and reliability of regression models was confirmed based on analysis including the elimination of outlier data points (based on measurements of Cook’s distance), the evaluation of the coefficient of multiple determination (R^2^) and the root mean square error (RMSE) and the correlations between fitted and observed data points (Supplementary Figure S2b), as well as through the visualization of residual plots, Cook’s distance plot, normal Q-Q plot and residual histogram [12] (Supplementary Figure S2c). Finally, an ANOVA lack of fit test was performed to confirm the selection of an appropriate model structure. Higher-order terms, such as three-drug interactions, are generally considered to make a negligible contribution to overall activity and are therefore not included in the analysis [56]. Regression models were used to eliminate compounds from the search, leading the subsequent search round, composed of the experimental exploration of a smaller subset of drug combinations defined by an OACD design and analysis based on regression modeling. Thus, the TGMO platform was applied in 3 sequential search rounds. The dose-optimization search (*Search 4*) was achieved with the final selection of 4 compounds performed in an expanded dose range. 

### 4.5. Three-Dimensional Cell Cultures

The efficacy of **C2** and corresponding monotherapies was evaluated in homotypic 786-O, seeded 3000 cells/well, and heterotypic three-dimensional (3D) models. The latter model consisted of 3000 786-O cells cocultured in a 1:1 ratio with 3000 NHDFα cells, and 600 freshly isolated HUVEC cells (corresponding to 10%). The cell mixture was supplemented with 2.5% matrigel (Corning matrigel, #354230) and 50 µL/well was seeded in round bottom 96-well plates with a cell-repellent surface (Cellstar^®^ 650970) by using an electronic multidispensing pipette at low speed. The plate was centrifuged at 400 × g at 4 °C. The whole procedure was performed on ice and materials were precooled to avoid polymerization of matrigel. The cocultured spheroids were incubated with drugs for 72 h. Cell viability was measured with the CellTiter-Glo^®^ 3D Cell Viability Assay (Promega, #G9681). Luminescent signals were measured with a luminescent plate reader (Biotek Cytation 3 with corresponding software at the standard settings). 

### 4.6. Two-Dimensional Cell Migration Assay

The 2-dimensional cell migration assay was performed in an endothelial cell scratch assay [57]. 786-O cells were seeded at a density of 3–3.5 × 10^4^ cells/well) cells in a 96-well cell culture plate and grown overnight. A scratch was made using a sterile scratch tool (Peira Scientific Instruments, Beerse, Belgium) and the treatment was administered immediately after. The scratch wounds were imaged using a Leica DMI3000 microscope (Leica, Rijswijk, The Netherlands) at ×5 magnification using Universal Grab 6.3 software (DCILabs, Keerbergen, Belgium). Imaging was performed at T = 0 h and several time points after scratching until T = 7 h. The size of the scratch was automatically quantified and analyzed using Scratch Assay 6.2 (DCILabs) software by calculating the absolute wound closure (initial minus final scratch surface) [58]. 

### 4.7. Immunofluorescence

Cells were seeded on glass in a 6-well plate with a density of 100,000 cells/well for 786-O, 786-OsunR and MDA435 150,000 cells/well for M14 cells the day prior to the experiment. To investigate the cause of multipolarity, cells were treated for 12H with DMSO 0.1% or **C2**, then with 1 µg/ml nocodazole (Sigma, Buchs, Switzerland) for 30 min. Normal medium was then added to the cells for 15 min before fixation. Cells were fixed with methanol at −20 °C for 6 min, rinsed with PBS, blocked with 3% BSA RT, then stained for 1 h RT. The following primary antibodies were used: human anti α-tubulin (1:50, this study), rabbit serum anti-γtubulin (1:2000, this study), and mouse anti-centrin (1:2000, Merck Millipore, clone 20H5, Buchs, Switzerland). The secondary antibodies (Invitrogen, Zug, Switzerland) used were Alexa Fluor 488 anti-mouse (A21202), Alexa Fluor 594 anti-rabbit (A11012), Alexa Fluor 568 anti-rabbit (A11036), and Alexa Fluor 647 anti-human (A21445), diluted 1:400 and incubated for 30 min at RT on cells. Coverslips were mounted with VECTASHIELD with DAPI (Vector Laboratories, Burlingame, CA, USA). Z-stack images were taken using an Olympus DeltaVision wide-field microscope (GE Healthcare, Chicago, IL, USA) with 60× 1.4 NA oil objective and recorded with a Coolsnap HQ2 CCD camera (Roper Scientific, Martinsried, Germany) and the Softworx software (GE Healthcare) with 0.2 μm spacing between stacks.

### 4.8. Mitosis Live Cell Imaging

To visually monitor the effect of the drug combination **C2** on 786-O cells, live-cell imaging was performed for 24 h at 37 °C on the T*i* widefield microscope (Nikon, Egg, Switzerland) equipped with an environmental chamber using either a 60× 1.3 NA or a 40× 1.3 oil objective and a CoolSNAP HQ camera (Roper Scientific, Tucson, AZ, USA). The sampling rate was 3 min with recording at each time point of 9 Z-stacks separated by 2 µm and 20 points were taken per condition. Two hours before the start of the movie, SiR-Tubulin was added on the cells (50 nM, SpiroChrome AG, Stein am Rhein, Switzerland) to stain microtubules, with Valspodar (4 µM) to keep the dye inside the cells. Time-lapse movies were analyzed using NIS Elements AR Software and cropped into figures using ImageJ FIJI software and Adobe Illustrator CC. 

### 4.9. Statistical Analysis

If not indicated differently, the data is presented as the mean of multiple independent experiments and error bars represent the standard error of the mean, unless otherwise specified. Significance was determined using the one-way or two-way ANOVA test with a post hoc multiple comparison test or a Student’s T-test (Graphpad Prism^®^). * *p* < 0.05 and ** *p* < 0.01 were considered statistically significant and are indicated versus the control or single-drug treatment groups, as specified in figure legends.

## 5. Conclusions

In summary, we discovered the low-dose synergistic drug combination **C2** that has the ability to inhibit multipolar spindle pole clustering in renal cell carcinoma 786-O and melanoma cells with high centrosome amplification. **C2** appears to selectively target a survival mechanism employed by cancer cells, promoting multipolar division and generation of nonproliferative daughter cells with compromised viability while showing negligible activity in nonmalignant cell types. Understanding the full mechanism of action of **C2** and testing its activity in various cancer types will allow analyzing whether the presence of multipolar spindles is a sole biomarker for **C2** treatment. 

## Figures and Tables

**Table 1:** Initial drug set used in the Therapeutically Guided Multidrug Optimization (TGMO) screen. Based on dose-response curves generated for each compound the ED_20_ dose was selected. Cell viability was measured using the CellTiter-Glo^®^ luminescence assay following a 72-hour incubation with drugs.

Drug	786-O		Target
	ED_50_ (μM)	ED_20_ (μM)	
CI-994	11.5	1	Class I HDACs
LBH-589	0.01	0.002	Class I and II HDACs
SAHA	3.7	1	Class I and II HDACs
tubacin	6	2	HDAC6
axitinib	11.5	0.2	VEGFRs, PDGFR
erlotinib	6.6	2	EGFR
BEZ-235	0.09	0.02	mTOR
dasatinib	0.1	0.05	SRC
VX-680	33	0.65	pan-Aurora Kinases
sorafenib	2.1	0.01	VEGFRs, PDGFR

## Supplementary Materials

The following are available online at https://www.mdpi.com/2072-6694/11/10/1612/s1. Figure S1: 786-O and HEK-293T dose-response curves for all drugs included in the TGMO-based screen. Figure S2: Overview of optimization procedure and results of first search round in TGMO. Figure S3: Results of drug combination optimization. Figure S4: Efficacy of non-optimized drug combinations screened in *Search 1* and composition of corresponding drug combinations. Figure S5: Anti-angiogenic activity of the optimized drug combinations. Figure S6: 786-O cell cycle and cell morphology are influenced by C2 treatment. Figure S7: Efficacy of optimized drug combinations on 786-O 2D migration. Figure S8: C2 does not affect HEK-293T cells. Figure S9: Sunitinib resistance induced in 786-O cells.
