# Supplementary material for: Identification of a Synergistic Multi-Drug Combination Active in Cancer Cells via the Prevention of Spindle Pole Clustering
Source: Cancers (Basel). 2019 Oct 22;11(10):1612. doi: 10.3390/cancers11101612 (PMC6826636; doi:10.3390/cancers11101612)
Supplement: Supplementary file 1 [file cancers-11-01612-s001.pdf]

Supplementary Material

Identification of a Synergistic Multi-Drug Combination Active in Cancer Cells via the Prevention of Spindle Pole Clustering

Supplementary Information

Drug Selection Justification

The most commonly used drugs to treat advanced renal cell carcinoma (RCC) are agents that target vascular endothelial growth factor (VEGF) receptors and those that inhibit mTOR. However, while most patients initially respond to these agents, with time they develop resistance and subsequent disease progression.

In this study, the set of ten drugs selected in this screen contains compounds that belong to tyrosine kinase inhibitors (TKI), histone deacetylase inhibitors (HDACI) and pan-Aurora kinases inhibitor. Six KI were included: axitinib, erlotinib, BEZ-235, dasatinib, VX-680, and sorafenib.

Axitinib (Inlyta®) is a second-generation TKI targeting selectively (VEGFR) 1, 2, and 3, and at lower affinity PDGFR-ß and c-KIT and is clinically used in the treatment of advanced RCC [1,2]. **Erlotinib** (Tarceva®) is used for treatment of nonsmall cell lung carcinoma to limit use to patients whose tumors have specific epidermal growth factor receptor (EGFR) mutations and for the treatment of patients with locally advanced, unresectable, or metastatic pancreatic cancer, in combination with gemcitabine. It has also been tested in treatment of advanced papillary RCC in combination with bevacizumab [3]. **BEZ-235** (Dactolisib®) is imidazoquinoline derivative acting as a PI3K and mTOR inhibitor that induces tumor cell apoptosis and growth inhibition in PI3K/mTOR-overexpressing tumor cells. It was tested in advanced RCC as monotherapy in phase1b clinical trial that was terminated due to toxicity constraints [4]. **Dasatinib**, a multikinase inhibitor which was originally approved for the treatment of Imatinib-Resistant Philadelphia Chromosome-Positive Leukemias as a result of its activity against the BCR-ABL tyrosine kinase. It has also been shown to act on the Src-YAP signaling axes, which would represent its major target in the treatment of solid tumors, including RCC [5]. **VX-680** (Tozasertib®) selectively targets the Aurora-A,-B,-C kinases, which is involved in cell cycle chromosome segregation and cytokinesis [6]. Inhibition of this kinase has been shown to induce cell cycle arrest and apoptosis [6] and it has undergone investigated in clinical trials for various solid tumors [7]. **Sorafenib** is a clinically approved multitarget TKI inhibiting various kinases, mainly Raf-1, B-Raf, and VEGFR-2. Sorafenib has shown clinical efficacy in the treatment of advanced and metastatic ccRCC [8,9], however with relatively high toxicity due to off-target effects.

Four histone deacetylase inhibitors are included in this screen, CI-994, LBH-589, SAHA, and tubacin. **CI-994** targets class I HDACs. It has been investigated in clinical trials in combination with various chemotherapeutic agents for the treatment of solid cancer with varying success [10,11]. **LBH-589** targets Class I and II HDACs and obtained FDA approval for the treatment of multiple myeloma in combination with bortezomib and dexamethasone after at least two other prior treatment regimens [12]. It has also been clinically examined for the treatment of RCC and other solid tumors alone [13] and in combination with bevacizumab and everolimus [14]. **SAHA** is also a Class I and II HDAC inhibitor approved for advanced primary cutaneous T-cell lymphoma [15]. It is also being clinically evaluated in various combination therapies for solid tumors, including with sorafenib [16]. Finally, **tubacin** (tubulin acetylation inducer) is a Class IIb HDAC inhibitor selective targeting HDAC 6 [17]. HDAC6 has been identified to play a role in the malignant progression of cancer has been identified as a relevant therapeutic target [18].

Orthogonal Array Composite Design (OACD)

In the s-FSC approach, experimental data points are selected and tested based on the design of experiment approach (DoE) (Figure 1a), and more specifically using a series of orthogonal array composite design (OACD) matrices. These matrices are based on the combination of a two-level fractional factorial design and a three-level orthogonal array design generating a resolution IV design matrix. The two-level fraction factorial design allows for the estimation of linear and bi-linear effects (i.e., single drug and 2-drug interaction coefficients), while the three-level orthogonal design allows for the estimation of linear and quadratic effects. Thus, the design allows for internal cross-validation of linear effects. The resulting design is a resolution IV matrix and allows for accurate screening for the most influential factors in the system based on accurate estimations of each factor's main effect (i.e., main effects are not aliased by other main effects or two-factor effects) [19], in addition to estimates of interaction and quadratic effects. Such matrices have previously been published in the literature [20] or can be developed de novo using appropriate statistical software (SAS Institute Inc., Statware Inc., SPSS Inc., etc.). Input requirements include the number of compounds to be screened (referred to as factors) and number of compound doses to be considered (referred to as levels, generally three in levels are applied in this methodology corresponding to two compound doses and a dosage of zero).

Endothelial–Pericyte Coculture Network Formation Assay

The assay was performed as previously described [21]. Briefly, adherent HUVEC and pericytes were washed in serum-free M199 medium and then labelled 30 min at 37 °C with either CellTracker Green CMFDA dye (HUVEC, CMFDA488, Life Technology/Molecular Probes: cat no. C-2925 stock: 10 mM; diluted to final 1 μM in M199 without FCS) or CellTracker Orange CMRA dye (pericytes, CMRA548, Life Technology cat no. C-34551; stock 5 mM; diluted to final 1 μM in M199 without FCS). angiogenesis micro slides (Ibidi GmbH, Gräfelfing, Germany) were incubated on ice and 12 μL of ice-cold growth factor-reduced Matrigel (Corning) was added to each well. Subsequently, complete M199 medium (50 μL) was added to each well and incubated for 45 minutes at 37 °C. 5000 HUVEC and 2500 pericytes were added to the polymerized Matrigel in each well, and cultured in complete M199 for 7–10 hours. Live cell imaging with NikonA1R (time-lapse, Z stack imaging) and Fiji/ImageJ with Angiogenesis Analyzer toolset was used for analysis.

Chorioallantoic Membrane (CAM) of the Chicken Embryo

An in vivo model of developmental angiogenesis was used to validate the anti-angiogenic activity of **C2** [22,23]. Fertilized chicken eggs were incubated in a hatching incubator at 37 °C with a relative humidity 65% [24]. On embryo development day (EDD) three, eggs were turned such that the narrow apex was facing up, a small hole was made in the top of the eggshell and closed with scotch tape. Eggs were returned to the incubated in a stationary position until EDD seven and the hole on the apex of the egg was expanded to approximately 3 cm in radius. A plastic ring was deposited on the CAM membrane and 20 μL treatments were administered within the ring. Treatment was performed twice, on EDD seven and eight, and the membranes were imaged via fluorescence angiograms on EDD nine using an epifluorescence microscope (Nikon AG, Eclipse FN1, Japan) coupled to pco.pixelfly. Fluorescein isothiocyanate dextran (FITC-dextran, 20 kDa, 20 μL, 25 mg/mL, Sigma-Aldrich) was injected intravascularly. To increase vascular contrast, 50 μL if black ink was injected (Pelikan, Witzikon, Switzerland) into the embryonic cavity. Image-based quantification using the number of branching points/mm^2 was performed using the ImageJ-based software [25].

Cell Cycle Analysis and Cell Death Induction by Flow Cytometry

Flow cytometry measurement of cellular DNA with the fluorochrome propidium iodide (PI) was used to identify cell cycle and apoptotic fractions. Cells were seeded in 6-well plates at a density of 20–40 × 10^3 cells/well and incubated for 24 hours. Medium or experimental condition was applied, and cells were incubated for an additional 72 hours. Both floating and attached cells were harvested, washed with PBS, resuspended and fixed with 70% ethanol, then incubated for 2 h at 4 °C. Cells were

stained with PI/RNAse staining solution (ThermoFisher Scientific, 1825102) for 30 min, in the dark, at room temperature. Cells were analyzed with the Attune NxT acoustic focusing cytometer (Life Technologies, Carlsbad, CA, USA) in the BL2 channel. Maximum excitation of PI bound to DNA is at 536 nm, and emission is at 617 nm. Apoptotic cells were defined as having subG1 DNA staining and quantified with Attune™ NxT Software v. 2.5 (Life Technologies).

F-Actin and Nuclear DAPI Cell Staining

786-O cells were seeded on glass in a 24-well plate with a density of 6000 cells/well. After 72 hours of treatment with optimized drug combination or monotherapies, the cells were fixed with 4% formaldehyde for 10 minutes at RT, washed twice and permeabilized with 0.1% Triton-X for 15 minutes. Cells were incubated at RT with Alexa Fluor 488 Phalloidin (A12379, Thermo Fisher) diluted 1:200 for 20 minutes to stain f-actin, washed twice and incubated at RT with 1 ug/mL Dapi (D9542, Sigma) for 5 minutes. After a final wash-step the glasses were imaged on the Biotek Cytation3 Imaging reader using a 10× objective.

Supplementary Figures

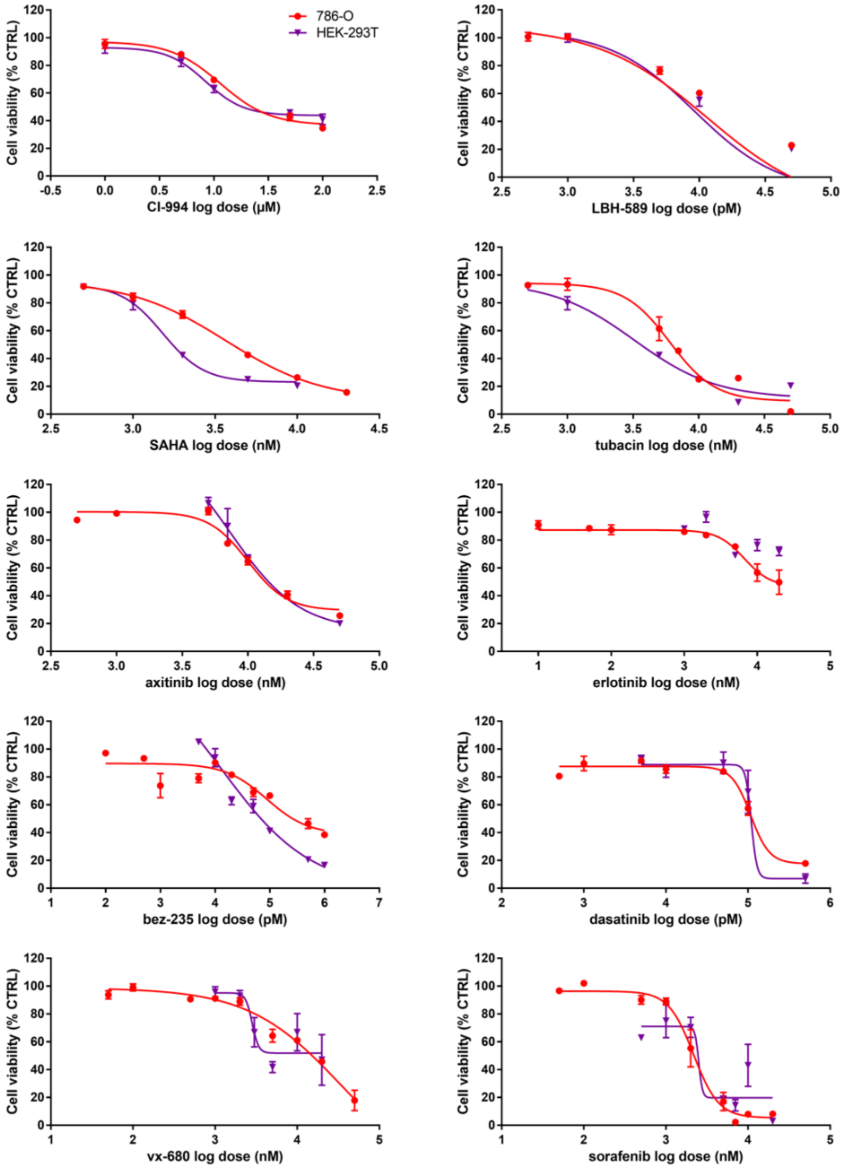

Figure S1. 786-O and HEK-293T dose-response curves for all drugs included in the Therapeutically Guided Multidrug Optimization (TGMO)-based screen. Using a four-parameter nonlinear fit of the

log-transformed doses for each compound (Graphpad Prism®), the ED_{20} concentration was calculated and selected as the high dose input in the TGMO-based screen.

a **Therapeutic window (TW) = (% CTRL non malignant cell viability) - (% CTRL cancer cell viability) =**

$$Int + \beta_1 D_1 + \beta_2 D_2 + \ldots \beta_{12} D_1 D_2 + \ldots \beta_{11} D_1 D_1 + \beta_{22} D_2 D_2 \ldots$$

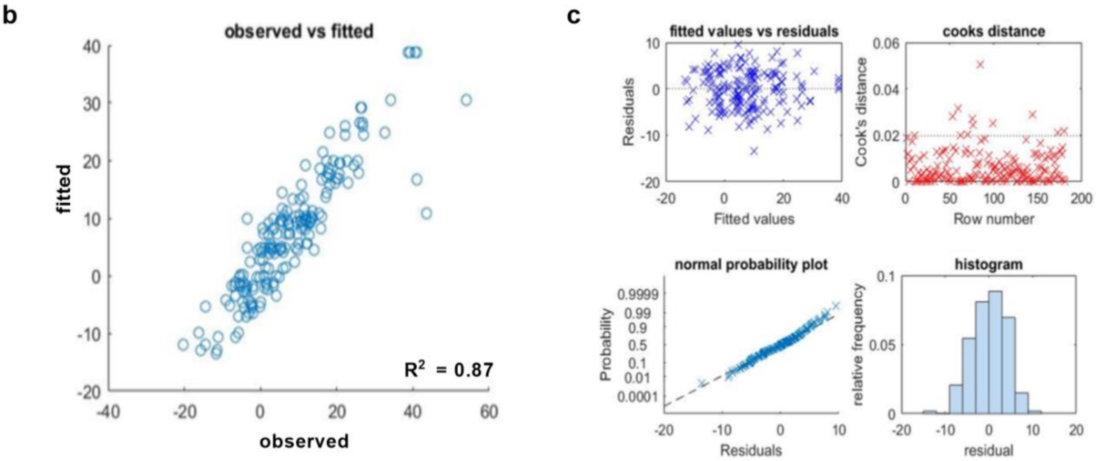

Figure S2. Overview of optimization procedure and results of the first search round in TGMO**.** The objective function defines the therapeutic window (TW) of each drug combination based on the difference in efficacy in cell viability inhibition between the malignant and nonmalignant cells and is modeled. (**b**) A plot of observed vs. fitted values with the R^2-value (coefficient of determination) as an indicator of goodness of fit. (**c**) Residual plots used for model analysis and the elimination of outlier data points if present (based on Cook's distance, generally defined as points with Cook's distance greater than three times the average Cook's distance of all data points). Residual plots include the observed vs. fitted data points, residual plot, Cook's distance plot, normal probability plot, and residual histogram.

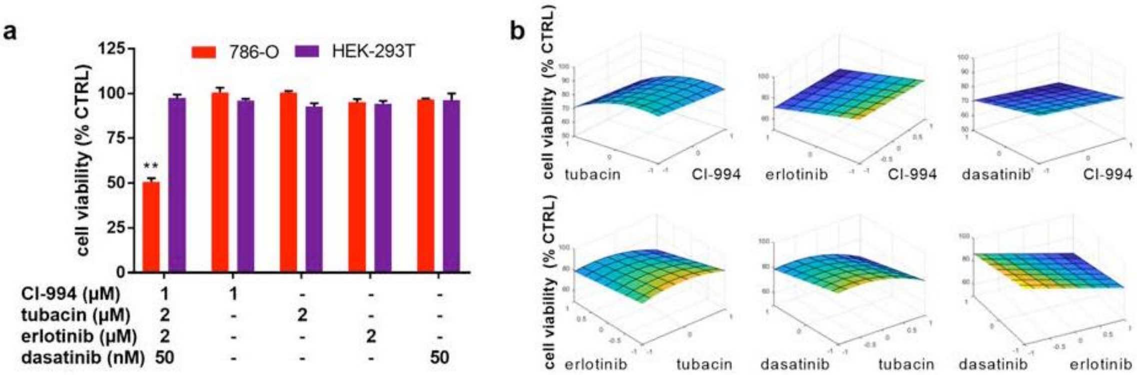

Figure S3. Results of drug combination optimization**.** Efficacy of the optimized drug combination **C1** and corresponding single drug therapies in 786-O cancer cells and the nonmalignant HEK-293T control cell line. Bars represent the mean of at least two independent experiments. ** $p < 0.01$ represent significance versus all corresponding single drug treatments as determined by a one-way ANOVA with post hoc Tukey's multiple comparison test. Error bars represent ± SEM. (**b**) Response surfaces showing the predicted viability of drug combinations when the dose of two compounds is varied and other compounds remain at a constant concentration. The curvature of the response surfaces indicates second-order interactions.

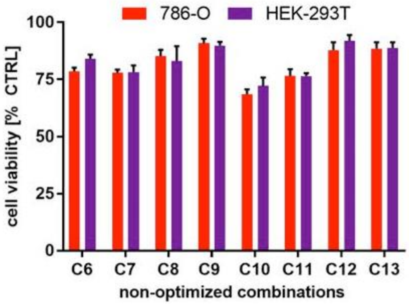

	C6	C7	C8	C9	C10	C11	C12	C13
CI-994 (µM)	X	X	X	X	-	-	-	-
LBH-589 (µM)	-	-	-	-	-	-	-	-
SAHA (µM)	X	X	-	-	X	X	-	-
tubacin (µM)	-	-	-	-	X	X	X	X
axitinib (µM)	-	-	X	X	-	-	X	X
erlotinib (µM)	X	-	X	-	X	-	X	-
BEZ-235 (µM)	-	-	-	-	-	-	-	-
dasatinib (µM)	-	X	X	-	X	-	-	X
VX-680 (µM)	-	X	-	X	-	X	-	X
sorafenib (µM)	X	-	-	X	-	X	X	-

x = ED_{20} dose
\- = no drug

Figure S4. Efficacy of non-optimized drug combinations screened in Search 1 and composition of corresponding drug combinations. The efficacy of non-optimized four-drug combinations derived from the screen in *Search 1* as part of the initial design matrix in the 786-O and non-malignant HEK-293T cells. Drug combinations have corresponding numbers of compounds compared to the final optimized combination and demonstrate low efficacy and selectivity of combinations prior to optimization.

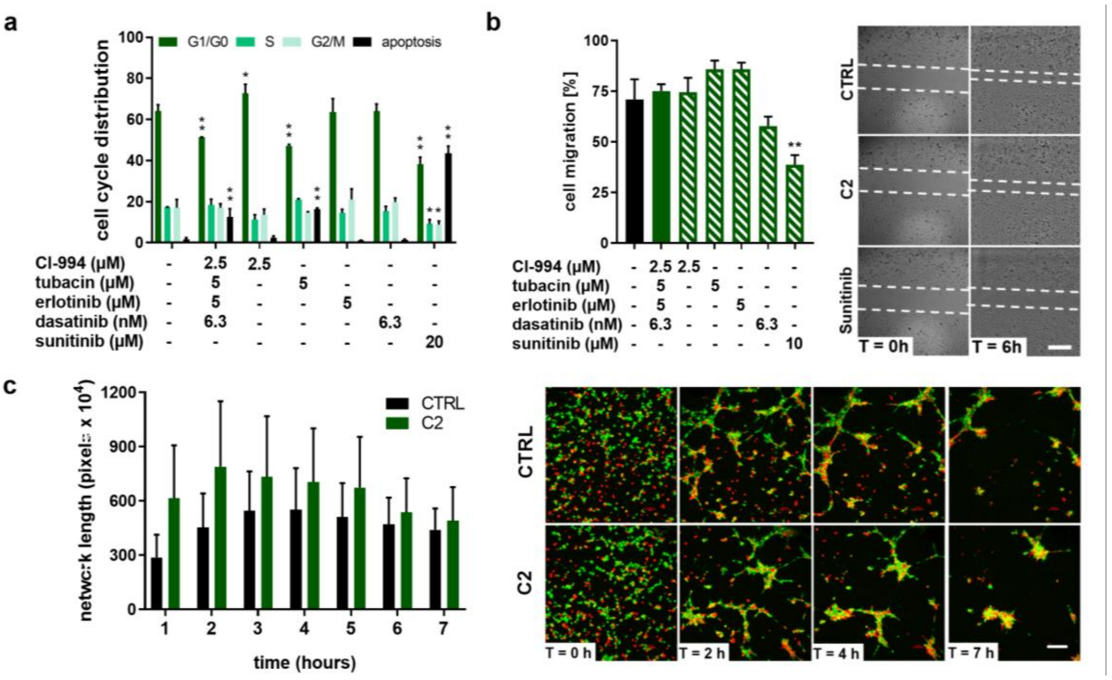

Figure S5. Anti-angiogenic activity of the optimized drug combinations. (**a**) Cell cycle analysis of ECRF24 cells treated with the 786-O optimized multidrug combinations (ODCs) for 72 h. Percentage of cells in G1, S, G2/M, or sub-G1 (apoptotic) is indicated compared to the 0.1% DMSO CTRL. Bars represent the mean of two independent experiments performed. * $p < 0.05$ and ** $p < 0.01$ represent significance versus CTRL determined by a two-way ANOVA with post hoc Dunnett's multiple comparison test. (**b**) 2D migration assay performed in ECRF24 cells. Bar graph shows the percentage of cell migration following the administration of a 'scratch' wound and 7 hours of incubation with the **C2** drug combination, corresponding monotherapies and sunitinib as a positive control. **$p < 0.01$ represents significance versus CTRL as determined by a one-way ANOVA with post hoc Dunnett's multiple comparison test from two independent experiments with n = 6. Representative images of the 2D migration assay are shown for CTRL, **C2**, and sunitinib positive control at the 6 h time point. Scale bar represents 400 µm for all images. (**c**) Matrigel coculture endothelial network formation assay with human umbilical vein endothelial cells (HUVEC) and human pericytes showing endothelial network formation over 7 hours. Confocal images of cells stained with cell tracker (HUVEC in green, pericytes in red) show the formation of an endothelial network in the CTRL treated wells. Quantification of the total endothelial network length in Fiji/ImageJ with Angiogenesis Analyzer shows no significant inhibition of HUVEC and pericyte network alignment and network formation from two independent experiments.

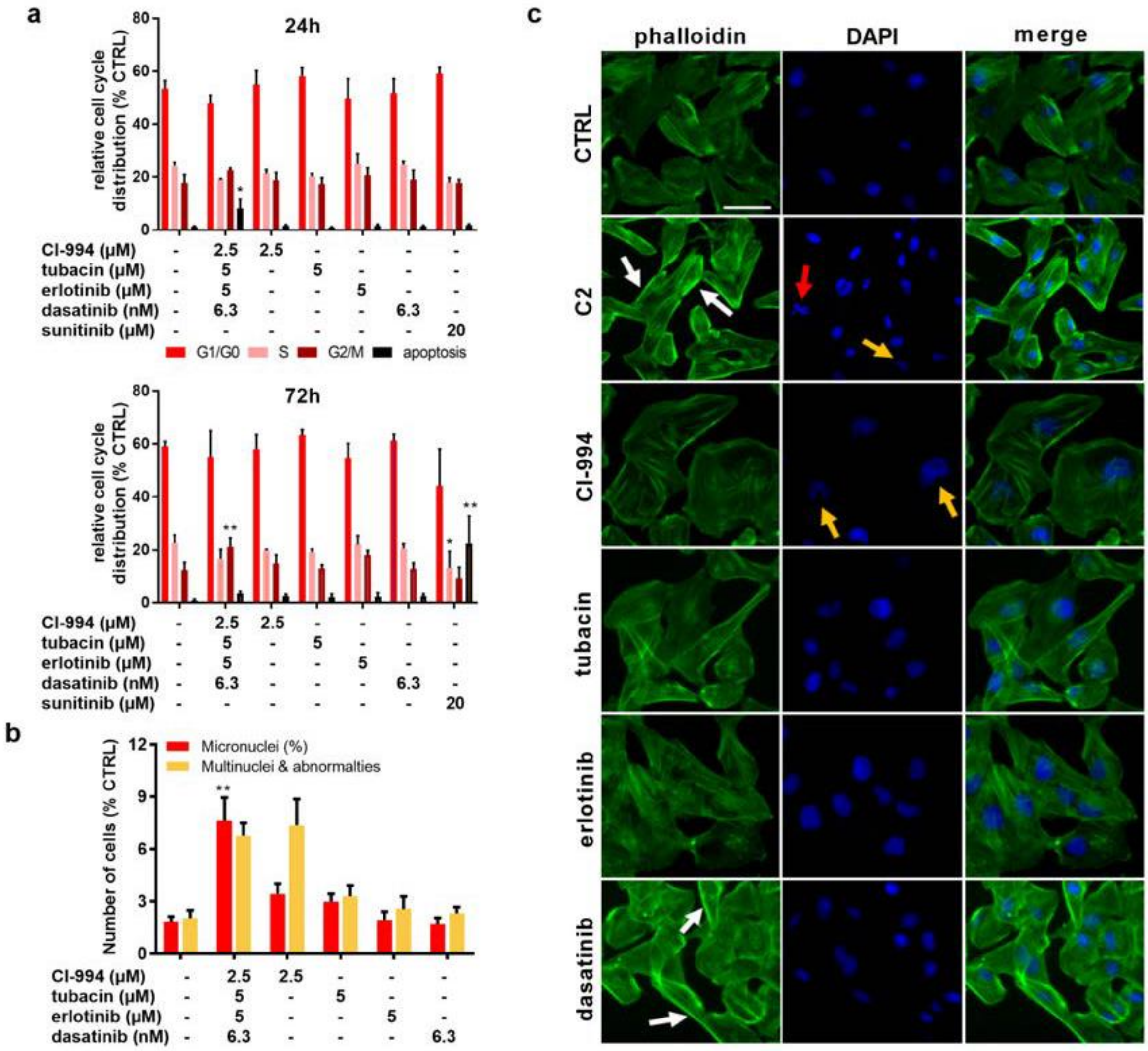

Figure S6. 786-O cell cycle and cell morphology are influenced by C2 treatment. (**a**) Cell cycle and cell death analysis in 786-O cells incubated with drugs for 24 or 72 hours, respectively. Bars represent the mean of at least two independent experiments. (**b**) Representative images of filamentous f-actin fluorescent staining (green, left column), nuclear 4',6-diamidino-2-phenylindole (DAPI) staining (blue, middle column), or merged images (right column) of 786-O cells. Cells were treated for 72 hours with the CTRL (0.1% DMSO), **C2** or corresponding monotherapies prior to fixation and staining. Arrows depict micronuclei (red), multi-nuclei/nucleic abnormalities (yellow), higher concentration of F-actin lining the cell membrane of 786-O cells (white). (**c**) Image-based quantification was performed on micronuclei and multi nuclei/nucleic abnormalities are presented as a percentage of the CTRL. Error bars represent the standard error of the mean (SEM). Significances represent * $p < 0.05$, ** $p < 0.01$ compared to the CTRL group as determined by a one-way ANOVA with posthoc Tukey's and Dunnett`s multiple comparison test, for **a** and **b** respectively. Scale bar represents 50 µm for all images.

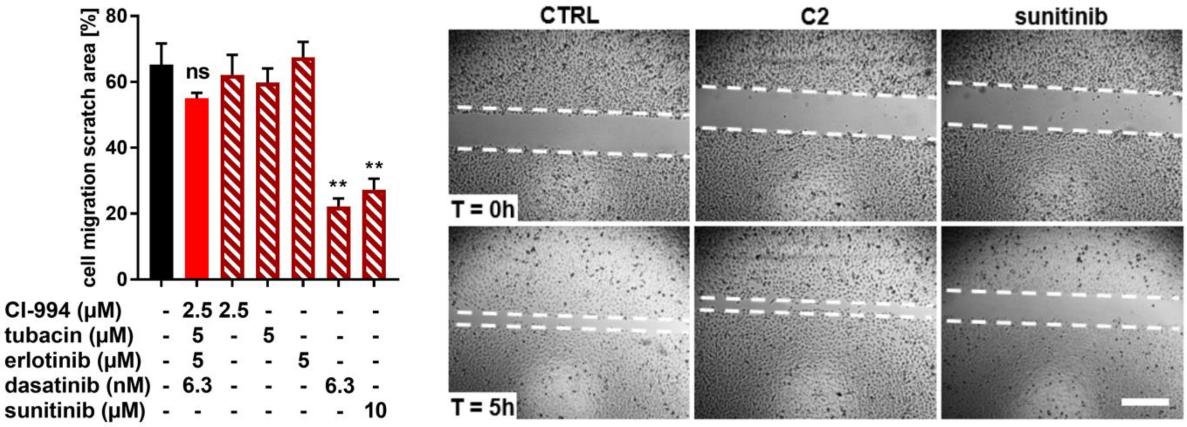

Figure S7. Efficacy of optimized drug combinations on 786-O 2D migration. 2D migration assay performed in 786-O cells. Bar graph shows the percentage of cell migration following the administration of a 'scratch' wound and 7 hours of incubation with the **C2** drug combination, corresponding monotherapies and sunitinib as a positive control. The final values are the mean of up to five independent experiments. ** $p < 0.001$ represents significance determined with a one-way ANOVA with post hoc Dunnett's multiple comparison test from two independent experiments with n = 6. Representative images of the 2D migration assay are shown for CTRL, **C2,** and sunitinib positive control at the 5 h time point. Scale bar represents 400 μm for all images.

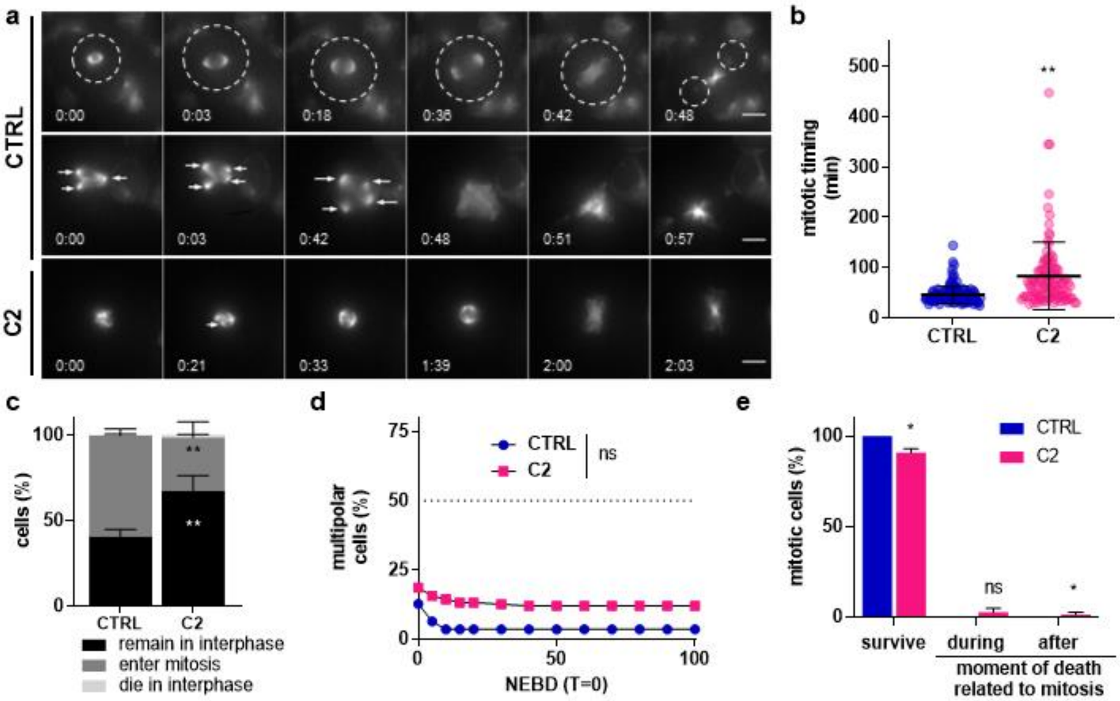

Figure S8. C2 does not affect HEK-293T cells. (**a**) Time-lapse images of CTRL and **C2**-treated HEK cells stained with SiR-Tubulin. Mitotic timing in h:mins using NEBD as T = 0. The arrows in the CTRL multipolar HEK cell indicate spindle poles. Scale bar = 10 μm. (**b**) Mitotic timing of HEK treated with CTRL or **C2**. ** $p < 0.0001$ compared to CTRL (0.1% DMSO) using a Mann–Whitney Test from N = 2 experiments with n = 111–131 cells. (**c**) Outcomes of HEK-293T cells treated with CTRL or **C2** during 24h movies. ** $p < 0.0001$ vs CTRL using Fisher's exact test, N = 2 experiments with n = 217–327 cells (**d**) Percentage of multipolar cells over time. **C2**-treated cells were compared to CTRL group using a two-way ANOVA with Sidak's multiple comparisons test from N = 2 experiments with n = 131–111 cells for CTRL and **C2**-treated cells. (**e**) Mitotic outcome of HEK-293T cells. * $p < 0.05$ vs CTRL using Fisher's exact test from N = 2 experiments with n = 131–111 cells for CTRL and **C2**-treated cells.

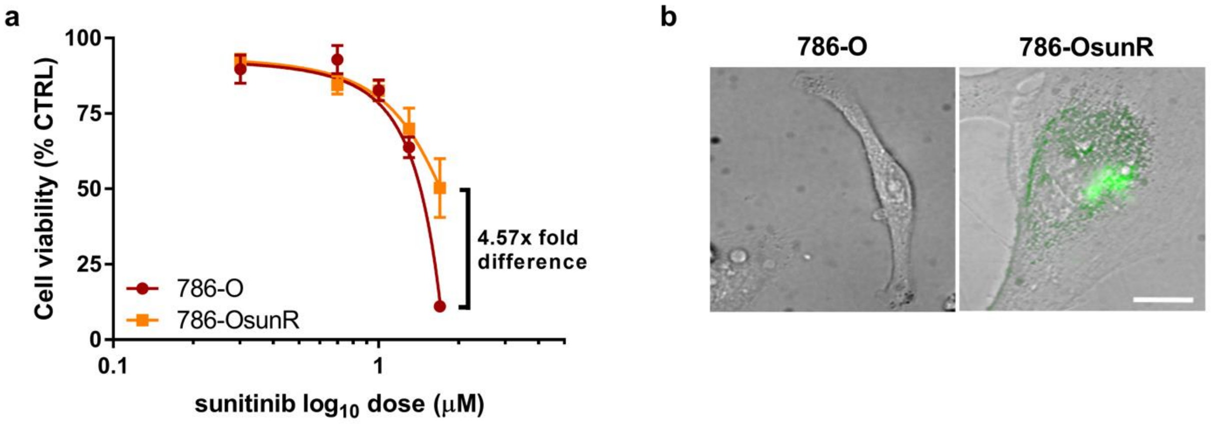

Figure S9. Sunitinib resistance induced in 786-O cells. (**a**) Sunitinib dose-response curves of naïve 786-O and 786-O cells chronically exposed to sunitinib (786-OsunR) treatment (1 μM) using the four-parameter non-linear fit of the log-transformed doses (Graphpad Prism®). Error bars represent ± SEM.* $p < 0.05$ and ** $p < 0.01$ represent significance between the RCC naïve and 786-OsunR cells determined with a two-way ANOVA with Sidak's multiple comparisons test of 3–4 independent experiments. (**b**) Representative bright-field and fluorescent images were taken at 540 nm demonstrating sunitinib accumulation in chronically treated cells. Images were acquired with 40x magnification. The scale bar represents 20 μm.
